# Multidrug-Resistant *Proteus mirabilis* and Other Gram-Negative Species Isolated from Native Egyptian Chicken Carcasses

**DOI:** 10.3390/tropicalmed9090217

**Published:** 2024-09-18

**Authors:** Bassant Ashraf El-Saeed, Hend Ali Elshebrawy, Amira Ibrahim Zakaria, Adel Abdelkhalek, Kálmán Imre, Adriana Morar, Viorel Herman, Khalid Ibrahim Sallam

**Affiliations:** 1 Faculty of Veterinary Medicine, Badr University in Cairo (BUC), Cairo 11829, Egypt; basant.ashraf@buc.edu.eg (B.A.E.-S.); adel.abdelkhalek@buc.edu.eg (A.A.); 2 Department of Food Hygiene, Safety, and Technology, Faculty of Veterinary Medicine, Mansoura University, Mansoura 35516, Egypt; hend_ali85@mans.edu.eg (H.A.E.); amera.zakaria@yahoo.com (A.I.Z.); 3 Department of Animal Production and Veterinary Public Health, Faculty of Veterinary Medicine, University of Life Sciences “King Mihai I” from Timişoara, 300645 Timișoara, Romania; adrianamo2001@yahoo.com; 4 Department of Infectious Diseases and Preventive Medicine, Faculty of Veterinary Medicine, University of Life Sciences “King Mihai I” from Timişoara, 300645 Timișoara, Romania; viorel.herman@fmvt.ro

**Keywords:** chicken meat, *Proteus mirabilis*, *Proteus vulgaris*, *Shigella*, multidrug-resistant

## Abstract

Poultry carcasses may be reservoirs for the zoonotic transmission of antimicrobial-resistant bacteria to humans and pose a major public health hazard. During the isolation of *Salmonella* from poultry and other foods, many of the presumptive typical *Salmonella* colonies on xylose lysine deoxycholate (XLD) agar were found to lack the *invA* gene, which is the specific target gene for *Salmonella* spp. Therefore, the current study aimed to estimate the prevalence and antimicrobial resistance profiles of extensively drug-resistant *invA*-negative non-*Salmonella* isolates recovered from native Egyptian chicken carcasses as presumptive *Salmonella* colonies on XLD agar. The non-*Salmonella* isolates were detected in 84% (126/150) of the examined native Egyptian chicken carcasses and classified into five genera, with prevalence rates of 64% (96/150), 14% (21/150), 6.7% (10/150), 3.3% (5/150), and 1.3% (2/150) for *Proteus*, *Citrobacter*, *Shigella*, *Pseudomonas*, and *Edwardsiella*, respectively. One hundred and ninety-five *invA*-negative, non-verified presumptive *Salmonella* isolates were recovered and classified at the species level into *Proteus mirabilis* (132/195; 67.7%), *Proteus vulgaris* (11/195; 5.6%), *Citrobacter freundii* (26/195; 13.3%), *Shigella flexneri* (8/195; 4.1%), *Shigella sonnei* (6/195; 3.1%), *Shigella dysenteriae* (3/195; 1.5%), *Pseudomonas fluorescens* (6/195; 3.1%), and *Edwardsiella tarda* (3/195; 1.5%). All (195/195; 100%) of these isolates showed resistance against cefaclor and fosfomycin. Additionally, these isolates showed high resistance rates of 98%, 92.8%, 89.7%, 89.2%, 89.2%, 86.7%, 80%, 78.5%, 74.4%, and 73.9% against cephalothin, azithromycin, vancomycin, nalidixic acid, tetracycline, sulfamethoxazole/trimethoprim, cefepime, gentamicin, cefotaxime, and ciprofloxacin, respectively. Interestingly, all (195/195; 100%) of the identified isolates were resistant to at least five antibiotics and exhibited an average MAR (multiple antibiotic resistance) index of 0.783. Furthermore, 73.9% of the examined isolates were classified as extensively drug-resistant, with an MAR index equal to 0.830. The high prevalence of extensively drug-resistant foodborne *Proteus*, *Citrobacter*, *Shigella*, *Pseudomonas*, and *Edwardsiella* isolated from native chicken carcasses poses a great hazard to public health and necessitates more monitoring and concern about the overuse and misuse of antibiotics in humans and animals. This study also recommends the strict implementation of GHP (good hygienic practices) and GMP (good manufacturing practices) in the chicken meat supply chain to protect consumer health.

## 1. Introduction

Chicken is one of the most consumed meats in the world [1]. The native chickens in Egypt are valued for their high nutritional quality, especially in terms of their high protein levels, low fat content, and high unsaturated fatty acid content, in addition to the absence of religious and cultural barriers to their consumption [2]. Their meat is richer in essential amino acids, making it a healthier choice for consumers. Native chicken production in Egypt contributes significantly to the country’s poultry sector, providing a key source of white meat. Consumption rates have increased in recent years, with native chicken being preferred for its taste and perceived health benefits. In Egypt, 1.2 billion birds are consumed annually. Furthermore, the Egyptian consumption of poultry meat grew from 1.01 million metric tons in 2016 to 1.59 million metric tons in 2023 [2,3,4]. Nonetheless, chicken meat is a leading reservoir for various pathogenic microorganisms such as *Salmonella* and *Campylobacter*, which can cause foodborne diseases in humans [5].

Globally, foodborne illnesses are becoming more common, increasing the financial burden on healthcare systems worldwide. After chickens have been slaughtered, their meat can become contaminated during preparation and processing with Gram-negative, non-spore-forming bacilli related to *Enterobacteriaceae* genera like *Shigella*, *Proteus*, *Citrobacter*, *Edwardsiella*, and *Pseudomonas. Enterobacteriaceae* are found mainly in the intestines of humans and other animals in addition to water, soil, and decomposing matter [1,2]. *Salmonella* is one of the most common foodborne pathogens, causing about 1.3 billion infections and 155,500 deaths worldwide yearly [3]. Consequently, the presence of *Salmonella* in food must be monitored continuously through appropriate analytical methods. Traditionally, bacteriological culturing methods are employed to isolate and identify *Salmonella* spp. XLD Agar is a selective culture medium used to isolate *Salmonella* and *Shigella* species from food and clinical samples [4].

*Proteus* spp. are opportunistic pathogens that primarily affect immunocompromised patients. The most prevalent pathogenic species is *P. mirabilis*, followed by *P. vulgaris*, which is less frequently isolated [5]. The bacterium *P. mirabilis* can cause wound infections, lower respiratory tract infections, urinary tract infections, and, rarely, sepsis and diarrhea [6]. Bacteria belonging to the genus *Proteus*, particularly *P. mirabilis*, have developed a severe pattern of antimicrobial resistance, especially to carbapenem drugs, which makes disease prevention and control more difficult [7]. Shigellosis is characterized by symptoms ranging from mild watery diarrhea to severe dysentery accompanied by systemic problems, such as an electrolyte imbalance, hemolytic uremic syndrome, and convulsions. The bacterium that produces Shiga toxin, *Shigella dysenteriae* subtype 1, is the deadliest and causes the most severe disease [8].

*Citrobacter* spp. are opportunistic human pathogens that can cause nosocomial infections, sporadic infections, and outbreaks [9]. The most frequently isolated *Citrobacter* species that causes diarrhea and other diseases is *C. freundii* [10]. Some strains of *C. freundii* have been linked to human food poisoning and diarrhea due to the presence of virulent factors such as heat-stable toxins, Shiga-like toxins, or virulent islands. Several *Citrobacter* species have been isolated from various foodstuffs, such as *C. freundii*, *C. braakii*, and *C. youngae* [11]. *Edwardsiella tarda* (*E. tarda*) is a Gram-negative bacterium that belongs to the *Enterobacteriaceae* family. The most common symptom of a bacterial infection with members of the *Edwardsiella* genus in humans is gastroenteritis, which seldom requires antibiotics. Although these organisms are perceived as infrequent foodborne pathogens or of dubious significance, it was reported that a patient infected with *Edwardsiella tarda* had gastroenteritis and required a prolonged antibiotic course [12]. *Pseudomonadaceae* members are Gram-negative, aerobic, non-spore-forming bacilli. *Pseudomonas fluorescens* and *P. aeruginosa* have been underestimated regarding their roles as foodborne pathogens. *Pseudomonas fluorescens* can infect humans and cause outbreaks of bacteremia [13]. *P. aeruginosa* can cause life-threatening infections such as meningitis, otitis media, urinary tract infections, and pneumonia. A few *Pseudomonas* species are susceptible to piperacillin, imipenem, ticarcillin, or ciprofloxacin, although the bulk of them are inherently resistant to penicillin and most related beta-lactam antibiotics [14].

Antimicrobial resistance is an emerging global problem, with poultry and poultry products serving as potential vehicles for multidrug-resistant bacteria, which humans can contract through direct contact with animals or their feces or by consuming or handling raw or undercooked meat [15,16]. The likelihood of bacterial populations developing antibiotic resistance increases with the number of antibiotics used, and there is mounting evidence that the widespread non-therapeutic application of antibiotics to animals has not only accelerated the emergence of resistant bacteria but also contributed to the development of a greater burden of chronic illness, heightened healthcare costs, and diminished effectiveness of antimicrobial drugs [7,16,17]. The current study was therefore designed to determine the prevalence and antimicrobial resistance profiles of of multidrug-resistant Gram-negative *invA*-negative non-*Salmonella* colonies involving *Proteus*, *Citrobacter*, *Shigella*, *Pseudomonas*, and *Edwardsiella* spp. isolated from native Egyptian chicken carcasses.

## 2. Materials and Methods

### 2.1. Collection and Preparation of Samples

Between July and November 2022, a total of 150 freshly dressed chicken carcass samples were obtained from several poultry shops in Mansoura, Egypt. The entire chicken carcasses were packaged individually in sterile polyethylene bags, transported under the cold conditions induced by an ice-packed container, and delivered in less than one hour to the Laboratory of Food Hygiene, Safety, and Technology Department of the Faculty of Veterinary Medicine of Mansoura University, Egypt, wherein they underwent bacteriological examination.

### 2.2. Isolation of Presumptive Typical Salmonella Colonies on XLD

The preparation, isolation, and identification of presumptive *Salmonella* colonies from chicken samples were carried out according to the guidelines provided by the Food Safety and Inspection Service of the United States Department of Agriculture [18]. Four hundred milliliters of sterile buffered peptone water (BPW; CM0509B; Oxoid Ltd., Basingstoke, UK) was poured into each of the sterile polyethylene bags containing the chicken carcasses. To make sure that both the interior and exterior sides of the chicken carcasses were washed properly with BPW, the bags were manually shaken for 5 min. Then, the rinse suspension was aseptically transferred into a sterile jar and incubated for 24 h at 37 °C. Subsequently, 1 mL of the pre-enrichment cultured Buffered Peptone Water (BPW, Oxoid) was aseptically added to 9 mL of Rappaport–Vassiliadis broth (RV; CM0669; Oxoid Ltd., Basingstoke, UK) and then incubated at 42 °C for 20–24 h. Following the incubation period, a loopful of enriched suspension was taken from the RV tubes that showed turbidity and streaked onto xylose lysine deoxycholate (XLD) agar (Oxoid, CM0469) plates as a specific solid medium for *Salmonella*. For each poultry sample, three XLD plates were seeded. The inoculated plates were incubated at 37 °C for 24 h. All of the 357 typical presumptive *Salmonella* colonies (which were pink with or without a black center) on XLD agar were held and preserved so that they could be subjected to biochemical and molecular identification.

### 2.3. Molecular Testing Conducted to Allow Differentiation between Salmonella and Other Gram-Negative Competitor Bacteria

The genomic DNA of the pink colonies with or without a black center, considered to constitute presumptive typical *Salmonella* isolates (n = 357) cultured on XLD, was extracted using QIAamp^®^ (Qiagen, Shenzhen, China) genomic DNA extraction kits based on the manufacturer’s prescript. PCR was carried out to determine whether the invasion gene (the *invA* gene), the specific marker gene for *Salmonella*, was present. Detection of the *invA* gene was performed using the forward (5′-ACAGTGCTCGTTTACGACCTGAAT-3′) and reverse (5′-AGACGACTGGTACTGATCGATAAT-3′) primer sequence sets, with an amplified band size of 244 bp [19]. The genomic DNA of the *Salmonella* Typhimurium reference strain obtained from the National Research Centre (NRC) in Dokki, Cairo, Egypt, was used as the positive control, and genomic DNA from *E. coli* K12 DH5α was used as the negative control. The *invA*-gene-negative colonies, which were not verified as *Salmonella*, were taken for subjection to biochemical examination and serological identification and to determine antibiotics resistance profiles.

### 2.4. Biochemical Identification

The *invA*-gene-negative non-*Salmonella* isolates (n = 228) were subjected to biochemical tests, including indole test, oxidation test, urease test, citrate test, methyl red test, Voges–Proskauer test, nitrate reduction test, triple-sugar iron (TSI) agar test, mannitol motility test, gelatin hydrolysis test, and a test concerning the fermentation of sugars (lactose, sucrose, dulcitol, salicin, arabinose, inositol, and xylose). All test vessels were incubated at 36 ± 1 °C, except for those used for the DNase (25 °C) and gelatin liquefaction (22 °C) tests. The results of the tests were examined after one to two days of incubation.

### 2.5. Serological Identification

Presumptive *Shigella* isolates were classified into serovars by performing slide agglutination test with polyvalent, somatic (O) antigen-grouping sera, followed by testing with monovalent antisera for the identification of specific serotypes according to the Manual of Clinical Microbiology [20]. Presumptive *Proteus* isolates were classified into serovars based on the O-specific polysaccharide chain (O antigen) using the agglutination technique [21]. Presumptive *Citrobacter* isolates were classified into serovars via the slide agglutination test according to O and H antisera [22,23]. Presumptive *Pseudomonas* isolates were classified into serovars by performing a slide agglutination test with polyvalent, somatic (O) antigen [24,25].

### 2.6. Antibiotic Susceptibility Testing for the Identified Isolates

The identified isolates were subsequently subjected to antimicrobial susceptibility testing according to the Kirby–Bauer disk diffusion technique on Mueller–Hinton agar (MH; CM0337; Oxoid Ltd., Basingstoke, UK) using guidelines established by the Clinical and Laboratory Standards Institute [26]. All 195 of the identified non-*Salmonella* isolates were tested against 14 antimicrobials belonging to nine antibiotic classes that involved cephalosporins (Cephalothin, KF—30 μg; Cefaclor, CEC—30 μg; Cefotaxime, CTX—30 μg; Cefepime, FEP—30 μg), macrolides (Azithromycin, AZM—15 μg), tetracyclines (Tetracycline, TE—30 μg), glycopeptides (Vancomycin, VA—30 μg), sulfonamides (Trimethoprim/Sulphamethoxazole, SXT—25 μg), quinolones (Nalidixic acid, NA—30 μg; Levofloxacin, LEV—5 μg; Ciprofloxacin, CIP—5 μg), carbapenems (Meropenem, MEM—10 μg), phosphonic antibiotics (Fosfomycin, FOS—50 μg), and aminoglycosides (Gentamicin, CN—10 μg). All antibiotic discs were purchased from Oxoid (Hampshire, England). After 24 h of incubation, the zone of inhibition was measured and interpreted as sensitive (S), intermediate (I), or resistant (R) according to CLSI [26].

Based on the antimicrobial resistance profiles, the tested isolates were categorized as follows: pan-drug-resistant (PDR) when they were resistant to all tested antimicrobials in all antimicrobial classes, extensively drug-resistant (XDR) when they were resistant to all tested antimicrobial classes except one or two classes, and multidrug-resistant (MDR) if they demonstrated resistance to at least one antimicrobial agent in three or more antimicrobial classes [27]. The MAR “multiple antibiotic resistance” index was calculated for all isolates by dividing the number of antimicrobials to which an isolate was resistant by the total number of antimicrobials tested [28]. An MAR index over 0.2 indicates a severe contamination risk and high misuse of antibiotics.

## 3. Results and Discussion

### 3.1. Prevalence of Gram-Negative Non-Salmonella Isolates in Native Egyptian Chicken Carcasses

Chicken carcasses are mainly contaminated by *Shigella*, *Proteus*, *Citrobacter*, and *Edwardsiella* species, which are frequently present in the digestive tract, lungs, skin, feathers, etc., during different processing procedures, such as bleeding, scalding, evisceration, chilling, storage, and so on [1]. Cultural legacies influence chicken consumption in Egypt since many consumers like to go to poultry retail outlets to choose chickens for slaughter; nonetheless, most poultry shops do not take proper sanitary measures during chicken slaughtering and processing.

**Figure 1:**
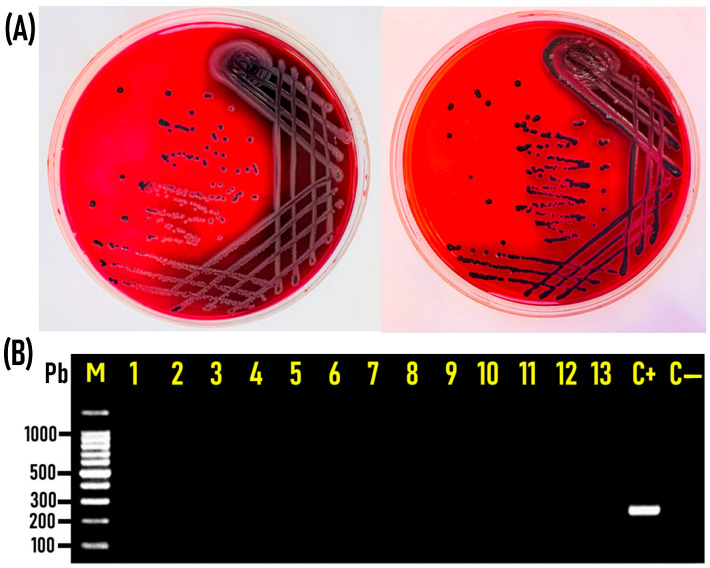
(**A**). Typical presumptive *Salmonella* colonies on xylose lysine deoxycholate (XLD) agar (in pink, with or without a black center) that were taken and subjected to PCR for the verification of *Salmonella* from non-*Salmonella* species. (**B**). Representative agarose gel electrophoresis image from the PCR assay for the detection of the *invA* (244 bp) gene specific for *Salmonella* in the genome prepared from the *Salmonella* presumptive colonies. M: DNA marker (100-bp gene ladder). Lanes 1–13: *invA*-gene-negative, indicating non-*Salmonella* isolates that were selected and subjected to further analysis. C+: positive control. C−: negative control. Eight microliters of the PCR product was separated via electrophoresis on 1.5% agarose gel and visualized under UV light.

**Figure 2:**
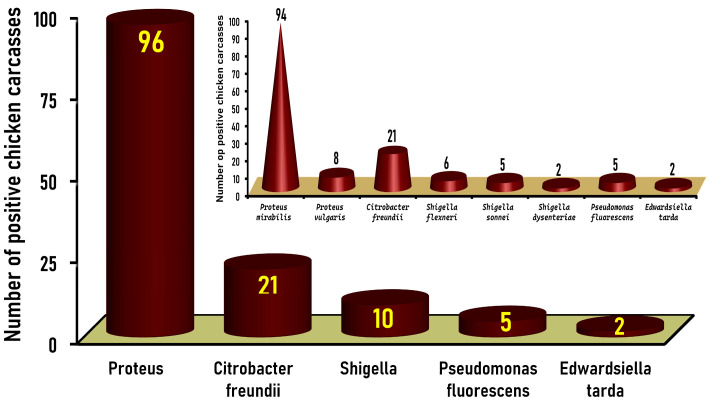
Prevalence of *Proteus mirabilis* and other Gram-negative non-*Salmonella* species in native Egyptian chicken carcasses tested (n = 150).

The pink colonies with or without black centers, considered presumptive typical *Salmonella* colonies (n = 357) on XLD agar (Figure 1A), were subjected to PCR tests to detect the *invA* gene, a specific marker gene for *Salmonella*. One hundred and twenty-nine isolates, recovered from 18% (27/150) of the chicken carcasses tested, were positive for the *invA* gene and confirmed to be *Salmonella* serovars, and these were further studied for their molecular characterization and antimicrobial resistance profiles [29]. The remaining isolates (n = 228) yielded negative results for the *invA* gene (Figure 1B) and were verified as non-*Salmonella* species. Thirty-three isolates of such non-*Salmonella* species were unidentified, while 195 colonies, recovered from 84% (126/150) of the native Egyptian chicken carcasses examined, were classified after biochemical and serological identification into five different genera. *Proteus*, *Citrobacter freundii*, *Shigella*, *Pseudomonas fluorescens*, and *Edwardsiella tarda* were detected in 96 (64%), 21 (14%), 10 (6.7%), 5 (3.3%), and 2 (1.3%) of the 150 chicken carcasses examined, respectively (Figure 2).

*Proteus* grows widely on XLD and can obfuscate the identification of *Salmonella* colonies [4]. *Proteus* is a growing public health concern that is found in various foods and poses a tremendous threat to public health [30]. Poultry and their products are considered the leading vehicles for the transmission of *Proteus* to humans via food of animal origin [31]. Of the 96 *Proteus*-positive chicken carcasses, 94 (62.7%) and 8 (5.3%) carcasses were positive for *Proteus mirabilis* and *Proteus vulgaris*, respectively (Figure 2 inset). Various prevalence rates for *Proteus* spp. in chicken meat have been reported worldwide. In Hong Kong, 85% (50/58) of fresh raw chicken carcass samples were positive for *Proteus mirabilis* [32]. In Indonesia, 51.7% (31/60) and 48.3% (29/60) of broiler chicken meat and backyard chicken meat, respectively, were contaminated by *Proteus* species [33]. In Belgium, *Proteus mirabilis* was isolated from 36.3% (29/80) of Belgian broiler carcasses [34], while in Pakistan, *P. mirabilis* was detected in 60% (9/15), 36% (4/11), and 33% (3/9) of the examined chicken liver, thigh, and wings, respectively [35]. Moreover, in regard to Lebanon, Barbour et al. [36] found a high recovery rate of 66% (33/50) for *Proteus mirabilis* in liver samples taken from individual broiler carcasses marketed by four major outlets. On the contrary, a low *Proteus* prevalence rate of 4% (4/100) was found in raw chicken breasts and thigh cuts in Ismailia City, Egypt [37]. Likewise, a low prevalence rate of 5% (6/100) was reported for *Proteus* species in broiler chicken carcasses tested in Iraq [38].

*Citrobacter freundii* was found in 14% (21/150) of native chicken carcasses tested (Figure 1). The *citrobacter* prevalence (14%) in chicken carcasses found in the current study is higher than the prevalence rate of 11.4% (11/70) determined in raw chicken meat samples in Nepal [39]. Likewise, 11.8% (13/110) of chicken carcasses examined in Iraq [40] were contaminated with *Citrobacter freundii*. On the other hand, high *Citrobacter* prevalence rates of 50% and 61.7% were reported in broiler chicken meat and backyard chicken meat, respectively, in Indonesia [33]. Also, a higher prevalence rate of 35.1% was found in chicken meat samples obtained from butcher shops in Yemen [41]. *Citrobacter* spp. are opportunistic pathogens that cause numerous infections of the gastrointestinal, urinary, and respiratory tracts; wounds; the intra-abdominal region; bone; the respiratory and biliary tracts (via calculi or blockages); surgical wounds; and the central nervous system. Additionally, they can settle in different tissues and organs [42]. *Citrobacter* can cause septicemia in patients with multiple predisposing factors, such as being immunocompromised; they can also cause meningitis, as well as lung infections in young children and newborns [43].

Of the 10 (6.7%) *Shigella*-positive chicken carcasses in the present study, 6 (4%), 5 (3.3%), and 2 (1.3%) carcasses were positive for *Shigella flexneri*, *Shigella sonnei*, and *Shigella dysenteriae*, respectively (Figure 2 inset). A previous study conducted in Egypt reported a meager prevalence rate of 0.6% (2/320) in chicken breast and thigh samples [44], while a low *Shigella* prevalence rate of 1% (1/100) was found in chicken breast and thigh cuts in Ismailia City, Egypt [37]. Nearly similar to our findings, a prevalence rate of 8% (7/87) was estimated in dressed chicken carcasses and chicken parts examined in Ghana [45], and also in Iraq [40], where a prevalence rate of 5.5% (6/110) was reported for *Shigella* species in chicken meat samples collected randomly from local markets in Baghdad. On the contrary, a very high prevalence rate of 97.3% (37/38) was recently detected for *Shigella* in chicken meat samples collected from 38 poultry butcher shops located in Jamshoro and Hyderabad in Pakistan [46]. High *Shigella* prevalence rates of 35% and 16.7% were also reported in broiler and backyard chicken meat in Indonesia [33], while a Shigella prevalence rate of 28% (14/50) was reported in chicken meat samples in India [47]. A lower rate of 11.9% was detected in chicken meat in Yemen [41]. On the contrary, Cetinkaya et al. [48] indicated that all (n = 168) of the broiler chicken parts (thighs, drumsticks, breasts wings, and necks) from Turkey tested in their study were negative for *Shigella. Shigella* is a leading foodborne pathogen, especially in Asia and Africa. Shigellosis is a severe illness in the majority of developing countries, accounting for at least 80 million instances of bloody diarrhea and 700,000 fatalities annually [49].

*Pseudomonas* has been described as one of the most ubiquitous bacterial genera in the world and a predominant genus in many foods, including chicken meat, where it primarily represents major food spoilage organisms [50]. *Pseudomonas fluorescens* was only detected in 3.3% (5/150) of chicken carcasses investigated in the present study (Figure 2). A previous study conducted in Egypt revealed a very high prevalence rate of 80% (40/50) for *Pseudomonas* spp. in fresh row chicken carcasses [51]. Likewise, a much higher prevalence rate of 92.5% was determined for the *Pseudomonas* genus in refrigerated chicken drumstick samples tested in Turkey [52]. In Saudi Arabia, although 69 (21.6%) of the 320 frozen chicken meat products examined (80 each of breasts, thighs, burgers, and nuggets) were positive for *Pseudomonas* spp., only 5 (1.6%) were positive for *Pseudomonas fluorescens* [53]. Additionally, a prevalence rate of 6% (6/100) was reported for *Pseudomonas* species in broiler chicken carcass samples tested in Iraq [38].

*Edwardsiella tarda* is categorized as a dangerous food- and waterborne infectious agent, increasing the risk of mortality for patients with liver cirrhosis [54]. In this study, *Edwardsiella tarda* was detected in 1.3% (2/150) of the chicken carcasses investigated (Figure 2). Similarly, 1.5% (3/203) of chicken meat sold at traditional markets in Indonesia was contaminated by *Edwardsiella*; nonetheless, high prevalence rates of 16.7% and 10% were detected for *Edwardsiella* in broiler chicken meat and backyard chicken meat examined in Indonesia [33].

### 3.2. Frequency Distribution of the Identified Gram-Negative Non-Salmonella Isolates Recovered from Native Egyptian Chicken Carcasses

**Figure 3:**
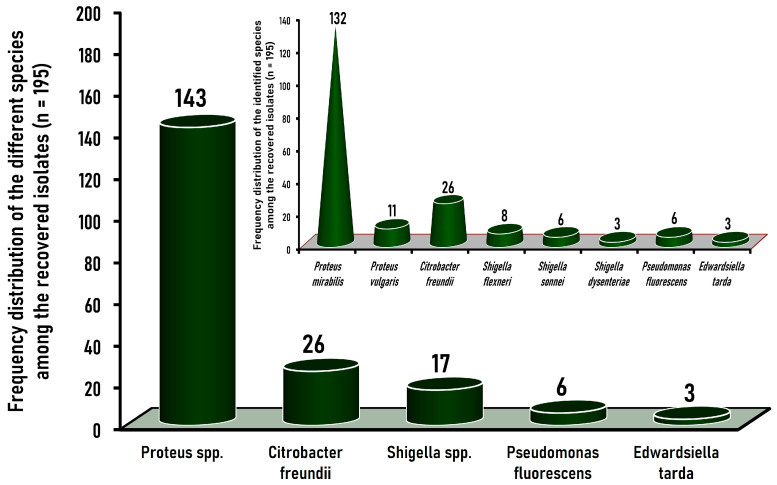
Frequency distribution of *Proteus mirabilis* and other species among the Gram-negative non-*Salmonella* isolates (n = 195) recovered from Egyptian chicken carcasses.

Among the 195 identified isolates recovered from chicken carcasses in the current study, *Proteus* species were the predominant Gram-negative organisms as they constituted 73.3% (143/195) of the isolates (Figure 3). Of these 143 *Proteus* isolates, 132 (92.3%) were serologically identified as *P. mirabilis*, while 11 (7.7%) were serotyped as *P. vulgaris* (Figure 3 inset). The rest of the isolates were classified according to the degrees of their prevalence as *Citrobacter freundii*, *Shigella*, *Pseudomonas fluorescens*, and *Edwardsiella tarda*, which were identified at incidences of 13.3% (26/195), 8.7% (17/195), 3.1% (6/195), and 1.5% (3/195) among the recovered isolates, respectively (Figure 3). The 17 *Shigella* isolates were further serotyped into three species, namely, *Shigella flexneri*, *Shigella sonnei*, and *Shigella dysenteriae*, which constituted 4.1% (8/195), 3.1% (6/195), and 1.5% (3/195) of the total Gram-negative isolates, respectively (Figure 3 inset).

The distribution and predominance of the Gram-negative isolates identified in the present study are inconsistent with those reported in many other publications that revealed that *Citrobacter* (not *Proteus*) was the most dominant species detected in chicken samples. For instance, Al-Asbahi [41] declared that among the 302 isolates recovered from chicken meat in Yemen, *Citrobacter* species were the predominant organisms since they constituted 35.1% (106/302) of the isolates, while *Proteus vulgaris*, *Shigella* spp., and *Proteus mirabilis* constituted 13.9% (42/302), 11.9% (36/302), and 8% (24/302) of the isolates, respectively. Likewise, *Citrobacter* species were the most predominant species identified among the 103 Gram-negative isolates recovered from 38 chicken meat samples examined in Nepal, where it constituted 44.7% (46/103), whereas *Proteus*, *Shigella*, and *Pseudomonas* were found at lower levels, namely, 18.4% (19/103), 3.9% (4/103), and 1.9% (2/103), respectively [55].

Although the *Proteus* species recovered from chicken vary among geographic regions, *Proteus mirabilis* and *Proteus vulgaris* were the most prevalent *Proteus* serovars recovered from chicken meat. In the current study, *P. mirabilis* was more predominant than *P. vulgaris*. On the other hand, *P. vulgaris* was the most common *Proteus* serovar in poultry meat examined in Yemen, with an incidence of 13.9%, while the incidence of *P. mirabilis* was 8% [41].

The 17 *Shigella* isolates identified in the present study were classified into three species, with *Shigella flexneri* being the most predominant, followed by *Shigella sonnei* and *Shigella dysenteriae*, which showed isolation frequency rates of 47.1% (8/17), 35.3% (6/17), and 17.6% (3/17), respectively. Similar to our findings concerning the frequency of *Shigella* isolation, Mberu [56] declared that *Shigella flexneri* was more predominant than *Shigella sonnei* as it constituted 67.7% (21/31) and 32.3% (10/31) of the 31 *Shigella* species isolated from chicken carcasses examined in Nigeria. On the contrary, 9 (64.2%) of the 14 *Shigella* isolates recovered from chicken meat in India were identified as *Shigella dysenteriae*, which was the most predominant species, while *Shigella flexneri* corresponded to only 5 (37.7%) isolates [47].

All (26/26, 100%) *Citrobacter* isolates recovered in the present study were serotyped into *Citrobacter freundii* (Figure 3), which constituted 13.3% (26/195) of the total Gram-negative isolates and was considered the second most predominant genera after *Proteus*. An approximately similar *C. freundii* incidence of 11.8% (13/110) was estimated among the 110 Gram-negative isolates isolated from chicken meat in Iraq [40], while a lower incidence amounting to 5.7% (26/459) was reported for *Citrobacter*, which was considered the fourth most dominant genus among the Gram-negative isolates recovered from retail meat samples (n = 310), including chicken meat samples (n = 103), in Japan [57].

In this study, the six (100%) *Pseudomonas* isolates recovered from chicken carcasses were confirmed to be *Pseudomonas fluorescens*, representing a low prevalence rate of 3.1% among the total Gram-negative isolates, while *P. aeruginosa* was not detected. Of the 69 *Pseudomonas* isolates recovered from chicken meat samples in Saudi Arabia, *Pseudomonas lundensis* was the most predominant, constituting 26.09% (18/69), while only 7.25% (5/69) of the pseudomonads were identified as *Pseudomonas fluorescens* [53]. Nonetheless, *P. fluorescens* was the most predominant species among the 325 molecularly confirmed isolates of the *Pseudomonas* genus recovered from retail chicken meat samples collected over 26 years in Norway [50]. Similarly, *Pseudomonas fluroscens* was the most predominant species, constituting 78.8% (78/99), among 99 isolates of the *Pseudomonas* genus obtained from chicken drumstick samples in Turkey, whereas *P. aeruginosa* was not detected [52].

In the current study, the *Edwardsiella* isolates (n = 3) recovered from native Egyptian chicken carcasses belonged to *Edwardsiella tarda*, with a low prevalence rate of 1.54% (3/195) among the total isolates (Figure 3). Similarly, in Egypt, 4 *Edwardsiella tarda* genera accounted for an incidence of 3.3% of the 121 isolates examined, comprising 10 different genera of Enterobacteriaceae recovered from 102 chickens [58]. In another study conducted in China, two *Edwardsiella tarda* isolates were isolated from 2 of the 30 duck liver samples examined [59].

The prevalence rates of Gram-negative microorganisms often vary according to differences in the geographical areas in which sampling was conducted, poultry species, farm hygiene, and sanitation levels, as well as the slaughtering methods employed for poultry. Generally, a high bacterial contamination level may be linked to unsanitary and unsatisfactory methods employed during chicken slaughter.

Ensuring health and hygiene in chicken meat production is crucial for preventing foodborne illnesses and safeguarding public health. The application of strict sanitation protocols in slaughterhouses and poultry-processing facilities, including regular cleaning and disinfection and appropriate practices for slaughter, dressing, and carcass preparation, can significantly reduce contamination risks posed by foodborne pathogens. Additionally, a continuous cold chain during distribution and retail, coupled with regular inspections and clean display areas, must be maintained to ensure the safety and high quality of poultry meat. Moreover, national and international food safety standards ensured through regular audits must be put in place to strengthen hygiene and safety throughout the poultry supply chain and ultimately reduce the likelihood of foodborne illnesses.

### 3.3. Antimicrobial Resistance of the Identified Species of Gram-Negative, Non-Salmonella Isolates Recovered from Native Egyptian Chicken Carcasses

Interestingly, all the Gram-negative non-*Salmonella* isolates recovered (100%, 195/195), comprising five genera of *Proteus*, *Citrobacter*, *Shigella*, *Pseudomonas*, and *Edwardsiella*, demonstrated resistance against cefaclor and fosfomycin (Table 1). Additionally, 98% (191/195), 92.8% (181/195), 89.7% (175/195), 89.2% (174/195), 89.2% (174/195), 86.7% (169/195), 80% (156/195), 78.5% (153/195), 74.4% (145/195), and 73.9% (144/195) of the isolates showed resistance against cephalothin, azithromycin, vancomycin, nalidixic acid, tetracycline, sulfamethoxazole/trimethoprim, cefepime, gentamicin, cefotaxime, and ciprofloxacin, respectively (Table 1). On the other hand, only 43.6% (85/195) of the isolates exhibited resistance against levofloxacin. Fortunately, it was noticed that 100% (195/195) of the isolates were susceptible to meropenem (Table 1).

In the veterinary field, antibiotics are used as growth promoters and to treat many infections. The spread and emergence of antimicrobial resistance have been widely associated with the misuse or indistinctive use of antibiotics in animal and human health settings. Gram-negative bacteria, including *Shigella*, *Proteus*, *Citrobacter*, *Pseudomonas*, and other less common bacteria such as *Edwardsiella*, are resistant to most antibiotics and increasingly becoming resistant to most available medications. These bacteria can develop novel strategies for resisting drugs and transferring their genetic materials to other bacteria to become drug-resistant. Gram-negative bacteria can acquire resistance to one or more leading classes of antibiotics, e.g., third- and fourth-generation cephalosporins (cefotaxime and ceftazidime), carbapenems (meropenem), fluoroquinolones (ciprofloxacin), aminoglycosides (gentamicin), tetracyclines, sulphonamides, and fosfomycin [60].

The resistance of all (100%) the isolates in the present study against cefaclor and fosfomycin is expected because such drugs are old medicines prescribed for a long time for treating multidrug-resistant *Enterobacteriaceae* infections. The high resistance incidences of the non-*Salmonella* isolates in the current study to cephalothin, nalidixic acid, azithromycin, vancomycin, tetracycline, sulfamethoxazole/trimethoprim, gentamicin, cefepime, cefotaxime, and ciprofloxacin suggest that these antibiotics are vastly used in veterinary medicine, giving a chance for the bacterial generations to develop resistance against these drugs.

All (100%) the *Proteus mirabilis* (n = 132) and *Proteus vulgaris* (n = 11) isolates exhibited resistance towards three antibiotics, namely, cefaclor, fosfomycin, and cephalothin, while 93.7%, 89.5%, 93%, 92.3%, 88.8%, 83.9%, 83.2%, 69.9%, 82.5%, and 51% of these isolates showed resistance to azithromycin, vancomycin, nalidixic acid, tetracycline, sulfamethoxazole/trimethoprim, cefepime, gentamicin, cefotaxime, ciprofloxacin, and levofloxacin, respectively, and no resistance to meropenem was detected (Table 1).

The high frequency of antimicrobial resistance exhibited by the *Proteus* isolates against the specified antimicrobials is higher than the resistance rate recorded for *Proteus* isolates from chickens in Indonesia against tetracycline, sulfamethoxazole/trimethoprim, ceftriaxone, ceftazidime, cefoxitin, gentamicin, and nalidixic acid [61]. In another study, Li et al. [62] reported that 98%, 98%, 14%, 10%, and 8% of *Proteus mirabilis* isolates isolated from broiler chicken farms in China exhibited resistance against ciprofloxacin, trimethoprim-sulfamethoxazole, cefepime, cefoxitin, and meropenem, respectively.

All the *Citrobacter freundii* isolates (n = 26) showed resistance against four antibiotics, namely, cefaclor, fosfomycin, azithromycin, and vancomycin (Table 1). Many isolates, however, showed resistance rates of 96.2%, 84.1%, 80.8%, 80.8%, 80.8%, 73.1%, and 57.7% for cefotaxime, cephalothin, nalidixic acid, tetracycline, sulfamethoxazole/ trimethoprim, cefepime, and gentamicin, respectively (Table 2). Lower rates of resistance were exhibited against tetracycline, sulfamethoxazole/trimethoprim, nalidixic acid, cefoxitin, gentamicin, erythromycin, and ciprofloxacin by *Citrobacter* isolates recovered from chicken meat examined in different countries, including Indonesia [60], India [63], and Nepal [39].

All (100%) *Shigella* isolates (n = 17) tested in the current study were resistant to five antibiotics, namely, cefaclor, fosfomycin, cephalothin, gentamicin, and ciprofloxacin. Moreover, 88.2%, 82.4%, 70.6%, 70.6%, 70.6%, and 70.6% of the isolates were resistant to nalidixic acid, cefotaxime, azithromycin, vancomycin, tetracycline, and sulfamethoxazole/trimethoprim, respectively (Table 1). Low resistance rates of 50%, 6.3%, and 50% against tetracycline, cefoxitin, and sulfamethoxazole/trimethoprim were observed for *Shigella* species recovered from chicken meat in Indonesia [61]. Conversely, in another study conducted in India, all (100%) of the *Shigella flexneri* isolates recovered from raw chicken meat were sensitive to azithromycin, cephalexin, ciprofloxacin, cefotaxime, gentamicin, and tetracycline, while all (100%) *Shigella dysenteriae* isolates were sensitive to cefotaxime and azithromycin [47].

In the current study, all the *Pseudomonas fluorescens* isolates (n = 6) exhibited resistance to eight antimicrobials, namely, cefaclor, fosfomycin, sulfamethoxazole/trimethoprim, cephalothin, azithromycin, vancomycin, tetracycline, and cefepime, while high resistance rates of 83.3%, 66.7%, 66.7%, and 50% were shown by *Pseudomonas fluorescens* against cefotaxime, nalidixic acid, ciprofloxacin, and levofloxacin, respectively (Table 1). Conversely, *Pseudomonas fluorescens* isolates collected from chicken meat samples in Saudi Arabia showed no resistance to cefepime, cefotaxime, cefoxitin, ceftazidime, ceftriaxone, ciprofloxacin, gentamicin, meropenem, or sulfamethoxazole/trimethoprim [53]. Likewise, *Pseudomonas* species isolated from chicken meat in Norway showed a resistance rate of 12.6% for meropenem [50].

Although *Edwardsiella* is not a common microorganism in chicken meat, the three isolates (100%) of *Edwardsiella tarda* identified in the present study exhibited resistance against 9 of the 14 antibiotics tested, namely, cefaclor, fosfomycin, cephalothin, azithromycin, vancomycin, tetracycline, sulfamethoxazole/trimethoprim, cefepime, and ciprofloxacin (Table 1), while a low resistance rate of 33.3% against nalidixic acid, cefotaxime, and levofloxacin was detected for *Edwardsiella* (Table 1). Likewise, *Edwardsiella* isolates obtained from chicken meat in Indonesia exhibited a high resistance rate of 66.7% towards tetracycline and sulfamethoxazole/trimethoprim along with a relatively low resistance rate of 33.3% against nalidixic acid and no resistance against gentamicin and cefoxitin [61].

### 3.4. Categorization of the Different Identified Gram-Negative (Non-Salmonella) Species Based on Their Antibiotic Resistance Profiles and Their Multiple Antibiotic Resistance (MAR) Index Values

Based on the results of our study, 73.9% (144/195) and 26.2% (51/195) of the examined isolates were classified according to their antibiotic resistance profiles into extensively drug-resistant and multidrug-resistant bacteria, respectively, while a low-level-drug-resistant profile category was not detected in this study (Table 2), which is an interesting finding indicating the excessive use of antibiotics in poultry farms in Egypt.

The antimicrobial resistance patterns of all the non-*Salmonella* isolates examined against the 14 antimicrobial agents revealed 25 different patterns, and 100% (195/195) of the isolates were resistant to at least 5 antibiotics (Table 3). Interestingly, 78% (103/132) of the *Proteus mirabilis* isolates, 72.7% (8/11) of the *Proteus vulgaris* isolates, 62.5% (5/8) of the *Shigella flexneri* isolates, 66.7% (4/6) of the *Shigella sonnei* isolates, 100% (3/3) of the *Shigella dysenteriae* isolates, 57.7% (15/26) of the *Citrobacter freundii* isolates, 83.3% (5/6) of the *Pseudomonas fluorescens* isolates, and (1/3) 33.3% of the *Edwardsiella tarda* isolates revealed resistance to all fourteen tested antimicrobial classes, except one or two, with an MAR index ranging between 0.714 and 0.929, with an average of 0.830 (Table 3).

The overall average “multiple antibiotic resistance” (MAR) index for the 195 isolates tested was 0.783, and 100% (195/195) of non-*Salmonella* isolates showed an MAR index of 0.357 or more (Table 2). An MAR index higher than 0.2 indicates the misuse and excessive use of antimicrobial agents on chicken farms [28]. The high prevalence of extensively drug-resistant Gram-negative non-*Salmonella* isolates recovered from Egyptian native chicken poses a tremendous hazard to public health and necessitates more monitoring. Therefore, it is essential to implement an extremely strict monitoring system to justify the use of antibiotics on chicken farms to safeguard public health against the spread of antimicrobial-resistant bacteria to humans through animal-derived food.

## 4. Conclusions

The current study reveals that Egyptian native chicken carcasses sold in Mansoura, Egypt, are heavily contaminated by extensively drug-resistant Gram-negative bacteria, posing a challenging threat to public health. *Proteus mirabilis* was the most frequently detected Gram-negative species, followed by *Citrobacter freundii*, *Proteus vulgaris*, *Shigella*, *Pseudomonas fluorescens*, and *Edwardsiella tarda*. Moreover, all the non-*salmonella* Gram-negative bacterial isolates were resistant to antimicrobial agents, with an MAR index ranging between 0.714 and 0.929, with an average of 0.830, sounding the alarm for the concerned health authorities to take strict measures and implement a surveillance system to limit the usage of antibiotics on poultry farms to safeguard the public from the spread of antimicrobial-resistant bacteria to humans through chicken meat consumption.

## Figures and Tables

**Table 1:** Antimicrobial resistance of the identified species of Gram-negative, non-*Salmonella* isolates (n = 195) recovered from native Egyptian chicken carcasses tested against the fourteen antimicrobials.

	*Proteus* (n = 143)		*Citrobacter* (n = 26)		*Shigella* (n = 17)		*Pseudomonas* (n = 6)		*Edwardsiella* (n = 3)		Total (n = 195)	
	No.	%	No.	%	No.	%	No.	%	No.	%	No.	%
Cefaclor (CEC)	143	100	26	100	17	100	6	100	3	100	195	100
Fosfomycin (FOS)	143	100	26	100	17	100	6	100	3	100	195	100
Cephalothin (KF)	143	100	22	84.6	17	100	6	100	3	100	191	98
Azithromycin (AZM)	134	93.7	26	100	12	70.6	6	100	3	100	181	92.8
Vancomycin (VA)	128	89.5	26	100	12	70.6	6	100	3	100	175	89.7
Nalidixic acid (NA)	133	93	21	80.8	15	88.2	4	66.7	1	33.3	174	89.2
Tetracycline (TE)	132	92.3	21	80.8	12	70.6	6	100	3	100	174	89.2
Sulfamethoxazole/Trimethoprim (SXT)	127	88.8	21	80.8	12	70.6	6	100	3	100	169	86.7
Cefepime (FEP)	120	83.9	19	73.1	8	47.1	6	100	3	100	156	80
Gentamicin (CN)	119	83.2	15	57.7	17	100	2	33.3	0	0.0	153	78.5
Cefotaxime (CTX)	100	69.9	25	96.2	14	82.4	5	83.3	1	33.3	145	74.4
Ciprofloxacin (CIP)	118	82.5	2	7.7	17	100	4	66.7	3	100	144	73.9
Levofloxacin (LEV)	73	51	0	0.0	8	47.1	3	50	1	33.3	85	43.6
Meropenem (MEM)	0	0.0	0	0.0	0	0.0	0	0.0	0	0.0	0	0.0

CEC, Cefaclor; FOS, Fosfomycin; KF Cephalothin; NA, Nalidixic acid; AZM, Azithromycin; VA, Vancomycin; TE, Tetracycline; SXT, Sulfamethoxazole/Trimethoprim; CN, Gentamicin; FEP, Cefepime; CTX, Cefotaxime; CIP, Ciprofloxacin; LEV, Levofloxacin.

**Table 2:** Antimicrobial resistance profile and multiple antibiotic resistance (MAR) indexes of the Gram-negative non-*Salmonella* isolates (n = 195) recovered from native Egyptian chicken carcasses.

Antimicrobial Resistance Patterns *	No. and (%) of Isolates	MAR Index	Resistance Profile	No. and (%) for Each Profile
CEC, FOS, KF, NA, AZM, VA, TE, SXT, CN, FEP, CTX, CIP, LEV	34 (17.4%)	0.929	Extensively drug-resistant	144 (73.85)
CEC, FOS, KF, NA, AZM, VA, TE, SXT, FEP, CTX, CIP, LEV	6 (3.1%)	0.857		
CEC, FOS, KF, NA, AZM, VA, TE, SXT, CN, CTX, CIP, LEV	5 (2.6%)	0.857		
CEC, FOS, KF, NA, AZM, VA, TE, SXT, CN, FEP, CTX, CIP	5 (2.6%)	0.857		
CEC, FOS, KF, NA, AZM, VA, TE, SXT, CN, FEP, CIP, LEV	15 (7.7%)	0.857		
CEC, FOS, KF, AZM, VA, TE, SXT, FEP, CTX, CIP, LEV	1 (0.5%)	0.786		
CEC, FOS, KF, NA, AZM, VA, TE, SXT, CN, FEP, CTX	25 (12.8%)	0.786		
CEC, FOS, KF, NA, AZM, VA, TE, CN, CTX, CIP, LEV	5 (2.6%)	0.786		
CEC, FOS, KF, NA, AZM, VA, TE, SXT, CN, CTX, CIP	14 (7.2%)	0.786		
CEC, FOS, KF, NA, VA, TE, SXT, CN, CTX, CIP, LEV	3 (1.5%)	0.786		
CEC, FOS, KF, NA, AZM, VA, TE, SXT, CN, FEP, CIP	20 (10.3%)	0.786		
CEC, FOS, KF, NA, AZM, VA, TE, SXT, CTX, CIP	1 (0.5%)	0.714		
CEC, FOS, KF, NA, AZM, VA, TE, SXT, CN, CTX	5 (2.6%)	0.714		
CEC, FOS, KF, NA, AZM, VA, TE, SXT, CN, FEP	5 (2.6%)	0.714		
CEC, FOS, KF, NA, VA, TE, SXT, FEP, CTX, CIP, LEV	4 (2.1%)	0.786	Multidrug-resistant	51 (26.15%)
CEC, FOS, KF, NA, VA, TE, CN, FEP, CTX, CIP, LEV	1 (0.5%)	0.786		
CEC, FOS, KF, NA, AZM, CN, FEP, CTX, CIP, LEV	10 (5.1%)	0.714		
CEC, FOS, KF, NA, AZM, TE, SXT, FEP, CTX, CIP	5 (2.6%)	0.714		
CEC, FOS, KF, NA, AZM, VA, TE, SXT, FEP, CTX	10 (5.1%)	0.714		
CEC, FOS, KF, NA, VA, SXT, CN, CTX, CIP, LEV	1 (5%)	0.714		
CEC, FOS, KF, AZM, VA, TE, SXT, FEP, CIP	10 (5.1%)	0.643		
CEC, FOS, KF, CN, FEP, CTX, CIP	4 (2.1%)	0.500		
CEC, FOS, KF, AZM, VA, CTX	1 (0.5%)	0.429		
CEC, FOS, KF, CN, FEP, CTX	1 (0.5%)	0.429		
CEC, FOS, AZM, VA, CTX	4 (2.1%)	0.357		
Overall Average MAR index = 0.783				

* CEC, Cefaclor; FOS, Fosfomycin; KF Cephalothin; NA, Nalidixic acid; AZM, Azithromycin; VA, Vancomycin; TE, Tetracycline; SXT, Sulfamethoxazole/Trimethoprim; CN, Gentamicin; FEP, Cefepime; CTX, Cefotaxime; CIP, Ciprofloxacin; LEV, Levofloxacin.

**Table 3:** Categorization of the different species of non-*Salmonella* isolates recovered from native Egyptian chicken carcasses according to their antimicrobial resistance profiles against the fourteen antimicrobials tested and their multiple antibiotic resistance (MAR) index values.

Serovars	Number of Isolates	* Antimicrobial Resistance Pattern	Antimicrobial Resistance Classes	MAR Index	Classification	
					Type of Resistance	No. and (%)
*Proteus mirabilis*	31	CEC, FOS, KF, NA, AZM, VA, TE, SXT, CN, FEP, CTX, CIP, LEV	Cephalosporin, phosphonic acid, Quinolone, Macrolides, Glycopeptide, Tetracyclines, Sulfonamides, Aminoglycosides, Fluoroquinolone	0.929	Extensively drug resistant	103 (78%)
	13	CEC, FOS, KF, NA, AZM, VA, TE, SXT, CN, FEP, CIP, LEV	Cephalosporin, phosphonic acid, Quinolone, Macrolides, Glycopeptide, Tetracyclines, Sulfonamides, Aminoglycosides, Fluoroquinolone	0.857		
	5	CEC, FOS, KF, NA, AZM, VA, TE, SXT, CN, FEP, CTX, CIP	Cephalosporin, phosphonic acid, Quinolone, Macrolides, Glycopeptide, Tetracyclines, Sulfonamides, Aminoglycosides, Fluoroquinolone	0.857		
	3	CEC, FOS, KF, NA, AZM, VA, TE, SXT, CN, CTX, CIP, LEV	Cephalosporin, phosphonic acid, Quinolone, Macrolides, Glycopeptide, Tetracyclines, Sulfonamides, Aminoglycosides, Fluoroquinolone	0.857		
	3	CEC, FOS, KF, NA, AZM, VA, TE, SXT, FEP, CTX, CIP, LEV	Cephalosporin, phosphonic acid, Quinolone, Macrolides, Glycopeptide, Tetracyclines, Sulfonamides, Fluoroquinolone	0.857		
	16	CEC, FOS, KF, NA, AZM, VA, TE, SXT, CN, FEP, CIP	Cephalosporin, phosphonic acid, Quinolone, Macrolides, Glycopeptide, Tetracyclines, Sulfonamides, Aminoglycosides, Fluoroquinolone	0.786		
	11	CEC, FOS, KF, NA, AZM, VA, TE, SXT, CN, FEP, CTX	Cephalosporin, phosphonic acid, Quinolone, Macrolides, Glycopeptide, Tetracyclines, Sulfonamides, Aminoglycosides	0.786		
	7	CEC, FOS, KF, NA, AZM, VA, TE, SXT, CN, CTX, CIP	Cephalosporin, phosphonic acid, Quinolone, Macrolides, Glycopeptide, Tetracyclines, Sulfonamides, Aminoglycosides, Fluoroquinolone	0.786		
	5	CEC, FOS, KF, NA, AZM, VA, TE, CN, CTX, CIP, LEV	Cephalosporin, phosphonic acid, Quinolone, Macrolides, Glycopeptide, Tetracyclines, Aminoglycosides, Fluoroquinolone	0.786		
	4	CEC, FOS, KF, NA, AZM, VA, TE, SXT, CN, CTX	Cephalosporin, phosphonic acid, Quinolone, Macrolides, Glycopeptide, Tetracyclines, Sulfonamides, Aminoglycosides	0.714		
	4	CEC, FOS, KF, NA, AZM, VA, TE, SXT, CN, FEP	Cephalosporin, phosphonic acid, Quinolone, Macrolides, Glycopeptide, Tetracyclines, Sulfonamides, Aminoglycosides	0.714		
	1	CEC, FOS, KF, NA, AZM, VA, TE, SXT, CTX, CIP	Cephalosporin, phosphonic acid, Quinolone, Macrolides, Glycopeptide, Tetracyclines, Sulfonamides, Fluoroquinolone	0.714		
	4	CEC, FOS, KF, NA, VA, TE, SXT, FEP, CTX, CIP, LEV	Cephalosporin, phosphonic acid, Quinolone, Glycopeptide, Tetracyclines, Sulfonamides, Fluoroquinolone	0.786	Multidrug-resistant	29 (22%)
	1	CEC, FOS, KF, NA, VA, TE, CN, FEP, CTX, CIP, LEV	Cephalosporin, phosphonic acid, Quinolone, Glycopeptide, Tetracyclines, Aminoglycosides, Fluoroquinolone	0.786		
	5	CEC, FOS, KF, NA, AZM, CN, FEP, CTX, CIP, LEV	Cephalosporin, phosphonic acid, Quinolone, Macrolides, Aminoglycosides, Fluoroquinolone	0.714		
	5	CEC, FOS, KF, NA, AZM, TE, SXT, FEP, CTX, CIP	Cephalosporin, phosphonic acid, Quinolone, Macrolides, Tetracyclines, Sulfonamides, Fluoroquinolone	0.714		
	4	CEC, FOS, KF, NA, AZM, VA, TE, SXT, FEP, CTX	Cephalosporin, phosphonic acid, Quinolone, Macrolides, Glycopeptide, Tetracyclines, Sulfonamides, Fluoroquinolone	0.714		
	1	CEC, FOS, KF, NA, VA, SXT, CN, CTX, CIP, LEV	Cephalosporin, phosphonic acid, Quinolone, Glycopeptide, Sulfonamides, Aminoglycosides, Fluoroquinolone	0.714		
	6	CEC, FOS, KF, AZM, VA, TE, SXT, FEP, CIP	Cephalosporin, phosphonic acid, Macrolides, Glycopeptide, Tetracyclines, Sulfonamides, Fluoroquinolone	0.643		
	2	CEC, FOS, KF, CN, FEP, CTX, CIP	Cephalosporin, phosphonic acid, Aminoglycosides, Fluoroquinolone	0.500		
	1	CEC, FOS, KF, CN, FEP, CTX	Cephalosporin, phosphonic acid, Aminoglycosides	0.429		
	Sum 132	Average MAR Index		0.806		
*Proteus vulgaris*	3	CEC, FOS, KF, NA, AZM, VA, TE, SXT, CN, FEP, CTX, CIP, LEV	Cephalosporin, phosphonic acid, Quinolone, Macrolides, Glycopeptide, Tetracyclines, Sulfonamides, Aminoglycosides, Fluoroquinolone	0.929	Extensively drug-resistant	8 (72.7%)
	2	CEC, FOS, KF, NA, AZM, VA, TE, SXT, CN, FEP, CIP, LEV	Cephalosporin, phosphonic acid, Quinolone, Macrolides, Glycopeptide, Tetracyclines, Sulfonamides, Aminoglycosides, Fluoroquinolone	0.857		
	1	CEC, FOS, KF, NA, AZM, VA, TE, SXT, CN, FEP, CIP	Cephalosporin, phosphonic acid, Quinolone, Macrolides, Glycopeptide, Tetracyclines, Sulfonamides, Aminoglycosides, Fluoroquinolone	0.786		
	1	CEC, FOS, KF, NA, AZM, VA, TE, SXT, CN, CTX, CIP	Cephalosporin, phosphonic acid, Quinolone, Macrolides, Glycopeptide, Tetracyclines, Sulfonamides, Aminoglycosides, Fluoroquinolone	0.786		
	1	CEC, FOS, KF, NA, AZM, VA, TE, SXT, CN, CTX	Cephalosporin, phosphonic acid, Quinolone, Macrolides, Glycopeptide, Tetracyclines, Sulfonamides, Aminoglycosides	0.714		
	2	CEC, FOS, KF, NA, AZM, CN, FEP, CTX, CIP, LEV	Cephalosporin, phosphonic acid, Quinolone, Macrolides, Aminoglycosides, Fluoroquinolone	0.714	Multidrug-resistant	3 (27.3%)
	1	CEC, FOS, KF, AZM, VA, TE, SXT, FEP, CIP	Cephalosporin, phosphonic acid, Macrolides, Glycopeptide, Tetracyclines, Sulfonamides, Fluoroquinolone	0.643		
	Sum 11	Average MAR Index		0.805		
*Shigella flexneri*	2	CEC, FOS, KF, NA, AZM, VA, TE, SXT, CN, CTX, CIP, LEV	Cephalosporin, phosphonic acid, Quinolone, Macrolides, Glycopeptide, Tetracyclines, Sulfonamides, Aminoglycosides, Fluoroquinolone	0.857	Extensively drug-resistant	5 (62.5%)
	2	CEC, FOS, KF, NA, AZM, VA, TE, SXT, CN, CTX, CIP	Cephalosporin, phosphonic acid, Quinolone, Macrolides, Glycopeptide, Tetracyclines, Sulfonamides, Aminoglycosides, Fluoroquinolone	0.786		
	1	CEC, FOS, KF, NA, VA, TE, SXT, CN, CTX, CIP, LEV	Cephalosporin, phosphonic acid, Quinolone, Glycopeptide, Tetracyclines, Sulfonamides, Aminoglycosides, Fluoroquinolone	0.786		
	2	CEC, FOS, KF, NA, AZM, CN, FEP, CTX, CIP, LEV	Cephalosporin, phosphonic acid, Quinolone, Macrolides, Aminoglycosides, Fluoroquinolone	0.714	Multidrug-resistant	3 (37.5%)
	1	CEC, FOS, KF, CN, FEP, CTX, CIP	Cephalosporin, phosphonic acid, Aminoglycosides, Fluoroquinolone	0.500		
	Sum 8	Average MAR Index		0.750		
*Shigella sonnei*	1	CEC, FOS, KF, NA, AZM, VA, TE, SXT, CN, CTX, CIP	Cephalosporin, phosphonic acid, Quinolone, Macrolides, Glycopeptide, Tetracyclines, Sulfonamides, Aminoglycosides, Fluoroquinolone	0.786	Extensively drug-resistant	4 (66.7%)
	1	CEC, FOS, KF, NA, VA, TE, SXT, CN, CTX, CIP, LEV	Cephalosporin, phosphonic acid, Quinolone, Glycopeptide, Tetracyclines, Sulfonamides, Aminoglycosides, Fluoroquinolone	0.786		
	2	CEC, FOS, KF, NA, AZM, VA, TE, SXT, CN, FEP, CIP	Cephalosporin, phosphonic acid, Quinolone, Macrolides, Glycopeptide, Tetracyclines, Sulfonamides, Aminoglycosides, Fluoroquinolone	0.786		
	1	CEC, FOS, KF, NA, AZM, CN, FEP, CTX, CIP, LEV	Cephalosporin, phosphonic acid, Quinolone, Macrolides, Aminoglycosides, Fluoroquinolone	0.714	Multidrug-resistant	2 (33.3%)
	1	CEC, FOS, KF, CN, FEP, CTX, CIP	Cephalosporin, phosphonic acid, Aminoglycosides, Fluoroquinolone	0.500		
	Sum 6	Average MAR Index		0.726		
*Shigella dysenteriae*	1	CEC, FOS, KF, NA, AZM, VA, TE, SXT, CN, CTX, CIP	Cephalosporin, phosphonic acid, Quinolone, Macrolides, Glycopeptide, Tetracyclines, Sulfonamides, Aminoglycosides, Fluoroquinolone	0.786	Extensively drug-resistant	3 (100%)
	1	CEC, FOS, KF, NA, VA, TE, SXT, CN, CTX, CIP, LEV	Cephalosporin, phosphonic acid, Quinolone, Glycopeptide, Tetracyclines, Sulfonamides, Aminoglycosides, Fluoroquinolone	0.786		
	1	CEC, FOS, KF, NA, AZM, VA, TE, SXT, CN, FEP, CIP	Cephalosporin, phosphonic acid, Quinolone, Macrolides, Glycopeptide, Tetracyclines, Sulfonamides, Aminoglycosides, Fluoroquinolone	0.786		
	Sum 3	Average MAR Index		0.786		
*Citrobacter freundii*	12	CEC, FOS, KF, NA, AZM, VA, TE, SXT, CN, FEP, CTX	Cephalosporin, phosphonic acid, Quinolone, Macrolides, Glycopeptide, Tetracyclines, Sulfonamides, Aminoglycosides	0.786	Extensively drug-resistant	15 (57.7%)
	2	CEC, FOS, KF, NA, AZM, VA, TE, SXT, CN, CTX, CIP	Cephalosporin, phosphonic acid, Quinolone, Macrolides, Glycopeptide, Tetracyclines, Sulfonamides, Aminoglycosides, Fluoroquinolone	0.786		
	1	CEC, FOS, KF, NA, AZM, VA, TE, SXT, CN, FEP	Cephalosporin, phosphonic acid, Quinolone, Macrolides, Glycopeptide, Tetracyclines, Sulfonamides, Aminoglycosides	0.714		
	6	CEC, FOS, KF, NA, AZM, VA, TE, SXT, FEP, CTX	Cephalosporin, phosphonic acid, Quinolone, Macrolides, Glycopeptide, Tetracyclines, Sulfonamides	0.714	Multidrug-resistant	11 (42.3%)
	1	CEC, FOS, KF, AZM, VA, CTX	Cephalosporin, phosphonic acid, Macrolides, Glycopeptide	0.429		
	4	CEC, FOS, AZM, VA, CTX	Cephalosporin, phosphonic acid, Macrolides, Glycopeptide	0.357		
	Sum 26	Average MAR Index		0.687		
*Pseudomonas fluorescens*	2	CEC, FOS, KF, NA, AZM, VA, TE, SXT, FEP, CTX, CIP, LEV	Cephalosporin, phosphonic acid, Quinolone, Macrolides, Glycopeptide, Tetracyclines, Sulfonamides, Fluoroquinolone	0.857	Extensively drug-resistant	5 (83.3%)
	2	CEC, FOS, KF, NA, AZM, VA, TE, SXT, CN, FEP, CTX	Cephalosporin, phosphonic acid, Quinolone, Macrolides, Glycopeptide, Tetracyclines, Sulfonamides, Aminoglycosides	0.786		
	1	CEC, FOS, KF, AZM, VA, TE, SXT, FEP, CTX, CIP, LEV	Cephalosporin, phosphonic acid, Macrolides, Glycopeptide, Tetracyclines, Sulfonamides, Fluoroquinolone	0.786		
	1	CEC, FOS, KF, AZM, VA, TE, SXT, FEP, CIP	Cephalosporin, phosphonic acid, Macrolides, Glycopeptide, Tetracyclines, Sulfonamides, Fluoroquinolone	0.643	Multidrug-resistant	1 (16.7%)
	Sum 6	Average MAR Index		0.786		
*Edwardsiella tarda*	1	CEC, FOS, KF, NA, AZM, VA, TE, SXT, FEP, CTX, CIP, LEV	Cephalosporin, phosphonic acid, Quinolone, Macrolides, Glycopeptide, Tetracyclines, Sulfonamides, Fluoroquinolone	0.857	Extensively drug resistant	1 (33.3%)
	2	CEC, FOS, KF, AZM, VA, TE, SXT, FEP, CIP	Cephalosporin, phosphonic acid, Macrolides, Glycopeptide, Tetracyclines, Sulfonamides, Fluoroquinolone	0.643	Multidrug-resistant	2 (66.7%)
	Sum 3	Average MAR Index		0.714		

* CEC, Cefaclor; FOS, Fosfomycin; KF Cephalothin; NA, Nalidixic acid; AZM, Azithromycin; VA, Vancomycin; TE, Tetracycline; SXT, Sulfamethoxazole/Trimethoprim; CN, Gentamicin; FEP, Cefepime; CTX, Cefotaxime; CIP, Ciprofloxacin; LEV, Levofloxacin.

## Data Availability

All data generated or analyzed during this study are included in the submitted version of this manuscript.

## References

[1] Parija S.C. (2023). Textbook of Microbiology and Immunology.

[2] Galal S. Annual Chicken Meat Production in Egypt 2010–2023. https://www.statista.com/statistics/1005988/egypt-chicken-meat-production/#:~:text=In%202023%2C%20chicken%20meat%20production,in%20the%20period%20under%20review.

[3] Sun T., Liu Y., Qin X., Aspridou Z., Zheng J., Wang X., Li Z., Dong Q. (2021). The prevalence and epidemiology of *Salmonella* in retail raw poultry meat in China: A systematic review and meta-analysis. Foods.

[4] Adzitey F., Huda N., Gulam R. (2011). Comparison of media for the isolation of *Salmonella* (XLD and Rambach) and *Listeria* species (ALOA and Palcam) in naturally contaminated duck samples. Internet J. Food Saf..

[5] Sun Y., Wen S., Zhao L., Xia Q., Pan Y., Liu H., Wei C., Chen H., Ge J., Wang H. (2020). Association among Biofilm Formation, virulence gene expression, and antibiotic resistance in *Proteus mirabilis* isolates from diarrhetic animals in Northeast China. BMC Vet. Res..

[6] Schaffer J.N., Pearson M.M. (2015). *Proteus mirabilis* and urinary tract infections. Microbiol. Spectr..

[7] Van Duin D., Doi Y. (2017). The global epidemiology of carbapenemase-producing Enterobacteriaceae. Virulence.

[8] Zaidi M.B., Estrada-García T. (2014). *Shigella*: A highly virulent and elusive pathogen. Curr. Trop. Med. Rep..

[9] Park Y.-J., Yu J.K., Lee S., Oh E.-J., Woo G.-J. (2007). Prevalence and diversity of qnr alleles in Ampc-producing *Enterobacter cloacae*, *Enterobacter aerogenes*, *Citrobacter freundii* and *Serratia marcescens*: A multicentre study from Korea. J. Antimicrob. Chemother..

[10] Samonis G., Karageorgopoulos D.E., Kofteridis D.P., Matthaiou D.K., Sidiropoulou V., Maraki S., Falagas M.E. (2009). *Citrobacter* infections in a general hospital: Characteristics and outcomes. Eur. J. Clin. Microbiol. Infect. Dis..

[11] Liu L., Lan R., Liu L., Wang Y., Zhang Y., Wang Y., Xu J. (2017). Antimicrobial resistance and cytotoxicity of *Citrobacter* spp. in Maanshan Anhui Province, China. Front. Microbiol..

[12] An L., Chan J.L., Nguyen M., Yang S., Deville J.G. (2023). Case report: Disseminated *Edwardsiella tarda* infection in an immunocompromised patient. Front. Cell Infect. Microbiol..

[13] Gershman M.D., Kennedy D.J., Noble-Wang J., Kim C., Gullion J., Kacica M., Jensen B., Pascoe N., Saiman L., McHale J. (2008). Multistate outbreak of *Pseudomonas fluorescens* bloodstream infection after exposure to contaminated heparinized saline flush prepared by a compounding pharmacy. Clin. Infect. Dis..

[14] Ryan K.J., Ray K.G. (2010). Sherris Medical Microbiology.

[15] Hedman H.D., Vasco K.A., Zhang L. (2020). A review of antimicrobial resistance in poultry farming within low-resource settings. Animals.

[16] Marshall B.M., Levy S.B. (2011). Food animals and antimicrobials: Impacts on human health. Clin. Microbiol. Rev..

[17] Njoga E.O., Nwanta J.A., Chah K.F. (2023). Detection of multidrug-resistant *Campylobacter* species from food-producing animals and humans in Nigeria: Public health implications and one health control measures. Comp. Immunol. Microbiol. Infect. Dis..

[18] USDA/FSIS Isolation and Identification of Salmonella from Meat, Poultry, Pasteurized Egg, Carcass, and Environmental Sponges (2023). United States Department of Agriculture Food Safety and Inspection Service MLG 4.13. https://www.fsis.usda.gov/sites/default/files/media_file/documents/MLG-4.13.pdf.

[19] Chiu C.H., Ou J.T. (1996). Rapid identification of *Salmonella* serovars in feces by specific detection of virulence genes, invA and spvC, by an enrichment broth culture-multiplex PCR combination assay. J. Clin. Microbiol..

[20] Carroll K.C., Pfaller M.A., Karlowsky J.A., Landry M.L., Mcadam A.J., Patel R., Pritt B.S. (2019). Manual of Clinical Microbiology.

[21] Knirel Y.A., Perepelov A.V., Kondakova A.N., Senchenkova S.N., Sidorczyk Z., Rozalski A., Kaca W. (2011). Structure and serology of O-antigens as the basis for classification of *Proteus* strains. Innate Immun..

[22] Lányi B. (1984). Lányi, B. 2 Biochemical and serological characterization of *Citrobacter*. Methods in Microbiology.

[23] Abbott S.L., Versalovic J., Carroll K.C., Funke G., Jorgensen J.H., Landry M.L., Warnock D.W. (2011). *Klebsiella*, *Enterobacter*, *Citrobacter*, *Serratia*, *Plesiomonas*, and other Enterobacteriaceae. Manual of Clinical Microbiology.

[24] Pitt T.L., Erdman Y.J. (1978). The specificity of agglutination reactions of *Pseudomonas aeruginosa* with O antisera. J. Med. Microbiol..

[25] Ansorg R., Knoche M. (1984). Determination of the O-serovars of *Pseudomonas aeruginosa* by slide coagglutination. Eur. J. Clin. Microbiol..

[26] CLSI “Clinical and Laboratory Standards Institute” (2020). Performance Standards for Antimicrobial Susceptibility Testing, M100.

[27] Magiorakos A.-P., Srinivasan A., Carey R.B., Carmeli Y., Falagas M.E., Giske C.G., Harbarth S., Hindler J.F., Kahlmeter G., Olsson-Liljequist B. (2012). Multidrug-resistant, extensively drug-resistant and pandrug-resistant bacteria: An international expert proposal for interim standard definitions for acquired resistance. Clin. Microbiol. Infect..

[28] Singh S., Yadav A.S., Singh S.M., Bharti P. (2010). Prevalence of *Salmonella* in chicken eggs collected from poultry farms and marketing channels and their antimicrobial resistance. Food Res. Int..

[29] El-Saeed B.A., Elshebrawy H.A., Zakaria A.I., Abdelkhalek A., Sallam K.I. (2024). Colistin-, cefepime-, and levofloxacin-resistant Salmonella enterica serovars isolated from Egyptian chicken carcasses. Ann. Clin. Microbiol. Antimicrob..

[30] Lei C.W., Zhang A.Y., Wang H.N., Liu B.H., Yang L.Q., Yang Y.Q. (2016). Characterization of SXT/R391 integrative and conjugative elements in *Proteus mirabilis* isolates from food-producing animals in China. Antimicrob. Agents Chemother..

[31] Firildak G., Asan A., Goren E. (2015). Chicken carcasses bacterial concentration at poultry slaughtering facilities. Asian J. Biol. Sci..

[32] Wong M.H.Y., Wan H.Y., Chen S. (2013). Characterization of multidrug-resistant *Proteus mirabilis* isolated from chicken carcasses. Foodborne Pathog. Dis..

[33] Yulistiani R., Praseptiangga D. (2019). Contamination level and prevalence of foodborne pathogen Enterobacteriaceae in broiler and backyard chicken meats sold at traditional markets in Surabaya, Indonesia. Malays. Appl. Biol..

[34] Yu Z., Joossens M., Van Den Abeele A.-M., Kerkhof P.-J., Houf K. (2021). Isolation, Characterization and antibiotic resistance of *Proteus mirabilis* from Belgian broiler carcasses at retail and human stool. Food Microbiol..

[35] Ishaq K. (2022). Occurrence and antimicrobial susceptibility of *Proteus mirabilis* from chicken carcass. PVJ.

[36] Barbour E.K., Hajj Z.G., Hamadeh S., Shaib H.A., Farran M.T., Araj G., Faroon O., Barbour K.E., Jirjis F., Azhar E. (2012). Comparison of phenotypic and virulence genes characteristics in human and chicken isolates of *Proteus mirabilis*. Pathog. Glob. Health..

[37] Afify S., Shaltout F., Mohammed I. (2020). Bacteriological profile of some raw chicken meat cuts in Ismailia City, Egypt. Benha Vet. Med. J..

[38] Noori T.E., Alwan M.J. (2016). Isolation and identification of zoonotic bacteria from poultry meat. Int. J. Adv. Res. Biol. Sci..

[39] Saud B., Paudel G., Khichaju S., Bajracharya D., Dhungana G., Awasthi M.S., Shrestha V. (2019). Multidrug-resistant bacteria from raw meat of buffalo and chicken, Nepal. Vet. Med. Int..

[40] Qader M.B.A., AlKhafaji M.H. (2019). Detection of bacterial contamination of imported chicken meat in Iraq. Iraqi J. Sci..

[41] Al-Asbahi A.A. (2022). Prevalence and Bacteriological Study of Gram-negative bacteria especially Citrobacter spp. in Poultry meat from Maeen Area- Sana’a, Yemen. J. Appl. Vet. Sci..

[42] Hashim M.H., AlKhafaji M.H. (2018). Isolation and identification of *Citrobacter freundii* from chicken meat samples using cultural and molecular techniques. Iraqi J. Sci..

[43] Sánchez-Códez M.I., Alonso-Ojembarrena A., Arca-Suárez J. (2018). Gramnegativos infrecuentes como agentes etiológicos de infecciones nosocomiales en una Unidad de Cuidados Intensivos Neonatales. Rev. Esp. Quim..

[44] Ahmed A.M., Shimamoto T. (2014). Isolation and molecular characterization of *Salmonella enterica*, *Escherichia coli* O157: H7 and *Shigella* spp. from meat and dairy products in Egypt. Int. J. Food Microbiol..

[45] Sackey B.A., Mensah P., Collison E., Sakyi-Dawson E. (2001). *Campylobacter*, *Salmonella*, *Shigella* and *Escherichia coli* in live and dressed poultry from metropolitan accra. Int. J. Food Microbiol..

[46] Tagar S., Qambrani N.A. (2023). Bacteriological quality assessment of poultry chicken meat and meat contact surfaces for the presence of targeted bacteria and determination of antibiotic resistance of *Salmonella* spp. in Pakistan. Food Control.

[47] Rabins L. (2021). Epidemiological study on *Shigella* from meat and its public health significance. Int. J. Curr. Microbiol. App. Sci..

[48] Cetinkaya F., Cibik R., Ece Soyutemiz G., Ozakin C., Kayali R., Levent B. (2008). *Shigella* and *Salmonella* contamination in various foodstuffs in Turkey. Food Control.

[49] WHO “World Health Organization” (2005). Shigellosis: Disease burden, epidemiology and case management. Relev. Epidemiol. Hebd..

[50] Heir E., Moen B., Åsli A.W., Sunde M., Langsrud S. (2021). Antibiotic resistance and phylogeny of *Pseudomonas* spp. isolated over three decades from chicken meat in the Norwegian food chain. Microorganisms.

[51] El-Aziz A. (2015). Detection of *Pseudomonas* spp. in chicken and fish sold in markets of Assiut City, Egypt. J. Food Qual. Hazards Control.

[52] Can H.Y. (2022). Investigation of *Pseudomonas* species in chicken drumstick samples. Kocatepe Vet. J..

[53] Elbehiry A., Marzouk E., Aldubaib M., Moussa I., Abalkhail A., Ibrahem M., Hamada M., Sindi W., Alzaben F., Almuzaini A.M. (2022). *Pseudomonas* species prevalence, protein analysis, and antibiotic resistance: An evolving public health challenge. AMB Expr..

[54] Samir S., Awad A., Younis G. (2021). Prevalence, virulence determinants and antimicrobial-resistant Profile of *Edwardsiella tarda* isolated from *Nile tilapia* (*Oreochromis niloticus*) in Egypt. AAVS.

[55] Shrestha A., Bajracharya A.M., Subedi H., Turha R.S., Kafle S., Sharma S., Neupane S., Chaudhary D.K. (2017). Multi-drug resistance and extended spectrum beta lactamase producing Gram negative bacteria from chicken meat in Bharatpur Metropolitan, Nepal. BMC Res. Notes.

[56] Mberu C. *Salmonella* and *Shigella* Species Associated with Broiler Chicken Meat and Their Susceptibility Patterns to Antimicrobials. Repository.mouau.edu.ng: 2021. https://repository.mouau.edu.ng/work/view/salmonella-and-shigella-species-associated-with-broiler-chicken-meat-and-their-susceptbility-patterns-to-antimicrobials-7-2.

[57] Odoi J.O., Takayanagi S., Sugiyama M., Usui M., Tamura Y., Asai T. (2021). prevalence of colistin-resistant bacteria among retail meats in Japan. Food Saf..

[58] Abd El-Tawab A.A., Selim A.O., Soliman A.M. (2018). Phenotypic and genotypic characterization of some bacterial isolates (*Escherichia coli*, *Klebsiella oxytoca*) from chickens. BVMJ.

[59] Wang X., Yan M., Wang Q., Ding L., Li F. (2012). Identification of *Edwardsiella tarda* isolated from duck and virulence genes detection. Afr. J. Microbiol. Res..

[60] CDC “Centers for Disease Control and Prevention” (2011). Gram-Negative Bacteria Infections in Healthcare Settings.

[61] Yulistiani R., Praseptiangga D., Supyani, Sudibya, Raharjo D., Shirakawa T. (2017). Prevalence of antibiotic-resistance *Enterobacteriaceae* strains isolated from chicken meat at traditional markets in Surabaya, Indonesia. IOP Conf. Ser. Mater. Sci. Eng..

[62] Li Z., Peng C., Zhang G., Shen Y., Zhang Y., Liu C., Liu M., Wang F. (2022). Prevalence and characteristics of multidrug-resistant *Proteus mirabilis* from broiler farms in Shandong Province, China. Poult. Sci..

[63] Moktan J.B., Venkataraman R., Shrestha Y. (2023). The prevalence of multidrug-resistant bacteria detected in poultry products in Mandya, India. Arch. Pharm. Pract..

